# Pathogenic mutations and overall survival in 3,084 patients with cancer: the Hellenic Cooperative Oncology Group Precision Medicine Initiative

**DOI:** 10.18632/oncotarget.27338

**Published:** 2020-01-07

**Authors:** Elena Fountzilas, Vassiliki Kotoula, Georgia-Angeliki Koliou, Eleni Giannoulatou, Helen Gogas, Christos Papadimitriou, Ioannis Tikas, Jianhua Zhang, Kyriaki Papadopoulou, Flora Zagouri, Christos Christodoulou, Angelos Koutras, Thomas Makatsoris, Sofia Chrisafi, Helena Linardou, Ioannis Varthalitis, George Papatsibas, Evangelia Razis, Pavlos Papakostas, Epaminontas Samantas, Gerasimos Aravantinos, Dimitrios Bafaloukos, Paris Kosmidis, Anna Koumarianou, Amanda Psyrri, Georgios Pentheroudakis, Dimitrios Pectasides, Andrew Futreal, George Fountzilas, Apostolia M. Tsimberidou

**Affiliations:** ^1^The University of Texas MD Anderson Cancer Center, Department of Investigational Cancer Therapeutics, Houston, TX, USA; ^2^Department of Pathology, Aristotle University of Thessaloniki, School of Health Sciences, Faculty of Medicine, Thessaloniki, Greece; ^3^Laboratory of Molecular Oncology, Hellenic Foundation for Cancer Research/Aristotle University of Thessaloniki, Thessaloniki, Greece; ^4^Section of Biostatistics, Hellenic Cooperative Oncology Group, Data Office, Athens, Greece; ^5^Victor Chang Cardiac Research Institute, Darlinghurst, NSW, Australia; ^6^The University of New South Wales, Kensington, NSW, Australia; ^7^First Department of Medicine, Laiko General Hospital, National and Kapodistrian University of Athens School of Medicine, Athens, Greece; ^8^Oncology Unit, Aretaieion Hospital, National and Kapodistrian University of Athens School of Medicine, Athens, Greece; ^9^Department of Genomic Medicine, The University of Texas MD Anderson Cancer Center, Houston, TX, USA; ^10^Department of Clinical Therapeutics, Alexandra Hospital, National and Kapodistrian University of Athens School of Medicine, Athens, Greece; ^11^Second Department of Medical Oncology, Metropolitan Hospital, Piraeus, Greece; ^12^Division of Oncology, Department of Medicine, University Hospital, University of Patras Medical School, Patras, Greece; ^13^Oncology Unit, Metropolitan Hospital, Piraeus, Greece; ^14^First Department of Medical Oncology, Henry Dunant Hospital, Athens, Greece; ^15^Oncology Department, University General Hospital of Larissa, Larissa, Greece; ^16^Third Department of Medical Oncology, Hygeia Hospital, Athens, Greece; ^17^Oncology Unit, Hippokration Hospital, Athens, Greece; ^18^Third Department of Medical Oncology, Agii Anargiri Cancer Hospital, Athens, Greece; ^19^Second Department of Medical Oncology, Agii Anargiri Cancer Hospital, Athens, Greece; ^20^First Department of Medical Oncology, Metropolitan Hospital, Piraeus, Greece; ^21^Second Department of Medical Oncology, Hygeia Hospital, Athens, Greece; ^22^Fourth Department of Internal Medicine, Attikon University Hospital, Athens, Greece; ^23^Section of Medical Oncology, Department of Internal Medicine, Attikon University Hospital, Faculty of Medicine, National and Kapodistrian University of Athens School of Medicine, Athens, Greece; ^24^Department of Medical Oncology, Medical School, University of Ioannina, Ioannina, Greece; ^25^Society for Study of Clonal Heterogeneity of Neoplasia (EMEKEN), Ioannina, Greece; ^26^Oncology Section, Second Department of Internal Medicine, Hippokration Hospital, Athens, Greece; ^27^The University of Texas MD Anderson Cancer Center, Department of Genomic Medicine, Houston, TX, USA; ^28^Aristotle University of Thessaloniki, Thessaloniki, Greece; ^29^Current address: Hellenic Cooperative Oncology Group, Athens, Greece

**Keywords:** actionable gene, next-generation sequencing, pathogenic mutation, precision oncology, prognosis

## Abstract

**Background:** We evaluated the association between pathogenic mutations and overall survival (OS) in patients with cancer referred to Hellenic Cooperative Oncology Group–affiliated Departments.

**Patients and methods:** Patients referred from 12/1980 to 1/2017 had molecular testing (for research) of archival tumor tissue collected at the time of first diagnosis (non-metastatic, 81%; metastatic, 19%). Tumor-specific gene panels (16-101 genes) were used to identify pathogenic mutations in clinically relevant genes. NGS genotyping was performed at the Laboratory of Molecular Oncology, Aristotle University of Thessaloniki. Annotation of mutations was performed at MD Anderson Cancer Center.

**Results:** We analyzed 3,084 patients (median age, 57 years; men, 22%) with sequencing data. Overall, 1,775 (58% of 3,084) patients had pathogenic mutations. The median follow-up was 7.52 years (95% CI, 7.39-7.61). In patients with non-metastatic tumors, after stratification by tumor type, increasing age, higher grade, and histology other than adenocarcinoma were associated with shorter OS. OS was also shorter in patients with pathogenic *TP53* (HR=1.36; p<0.001), *MLL3* (HR=1.64; p=0.005), and *BRCA1* (HR=1.46; p=0.047) mutations compared to wild-type genes. In multivariate analyses, independent prognostic factors predicting shorter OS were pathogenic mutations in *TP53* (HR=1.37, p=0.002) and *MLL3* (HR=1.50, p=0.027); increasing age (HR=1.02, p<0.001); and increasing grade (HR=1.46, p<0.001). In patients with metastatic cancer, older age and higher grade were associated with shorter OS and maintained their independent prognostic significance (increasing age, HR=1.03, p<0.001 and higher grade, HR=1.73, p<0.001).

**Conclusions:** Analysis of molecular data reveals prognostic biomarkers, regardless of tissue or organ of origin to improve patient management.

## INTRODUCTION

In the current era of precision medicine, recent advances in high-throughput technologies have enabled DNA sequencing in a timely, cost-effective, and non-labor-intensive manner. Next-generation sequencing (NGS) has facilited the identification of several molecular alterations that are being used in routine clinical cancer care as biomarkers to improve diagnostic accuracy, assessment of prognosis, and prediction of benefit from specific treatments [1–3]. Additionally, tumor molecular profiling has provided key insights into the mechanisms of tumorigenesis [4–6]. However, the clinical implications of these molecular alterations across tumor types remain to be fully elucidated.

In 1997, the Hellenic Cooperative Oncology Group (HeCOG) initiated a program to prospectively collect formalin-fixed, paraffin-embedded (FFPE) tumor tissue from patients referred to the affiliated Departments of Medical Oncology for assessment and treatment. HeCOG’s tumor repository now comprises of these samples, along with retrospectively collected tissue in selected cases. Matched germline DNA was also collected when possible. Protocols for the use of tumor tissue for research purposes were approved by the bioethics committees of the participating institutions. The clinical database of HeCOG was initiated in 1990.

HeCOG has previously investigated the genomic profiles of different tumor types using cancer-specific panels designed on the basis of available published data [7–15]. From 2013 to 2017, NGS was performed in the Laboratory of Molecular Oncology, Hellenic Foundation for Cancer Research, Aristotle University of Thessaloniki, to assess clinically relevant molecular alterations in patients with cancer. Research cohorts included patients with adequate tumor tissue for NGS and annotated clinical data. The studies found that mutations in breast and colorectal tumors were associated with survival [7, 9, 12, 16], and mutations in nasopharyngeal and biliary tumors occurred in clinically relevant genes [13, 15].

In the current analysis, we included all patients with informative NGS data from tumors that had been retrieved from the HeCOG repository and explored the association between pathogenic mutations across tumor types and overall survival (OS). We also examined the independent prognostic significance of frequently mutated genes in patients with non-metastatic versus metastatic cancer.

## RESULTS

### Patient characteristics

From 12/1980 to 12/2018, >40,000 patients were registered in the HeCOG clinical database (including 10,874 breast, 7,528 colorectal, 3,689 ovarian, 1,220 gastric, 504 glioma, 503 pancreatic, 436 nasopharyngeal, and 88 biliary). NGS was performed using tumor samples from 3,084 patients with the eight tumor types of interest. The median patient age was 56.7 years (range, 18.1-94.4 years), and 686 (22%) were men. The most common tumor types were breast (n=1,839, 60%) and colorectal (n=524, 17%). Overall, 2,430 (81%) patients were diagnosed with non-metastatic (stage I-III) disease and 569 (19%) with metastatic (stage IV) disease. Gliomas were included in metastatic tumors. The most common histology was adenocarcinoma (n=2,812; 91%). Patient baseline characteristics are shown in Table 1.

**Table 1:** Baseline characteristics

	All patients (N=3,084)	Patients with pathogenic mutations (N=1,775)	Patients without pathogenic mutations (N=1,309)	p-value
**Age^†^ (Median, Range)**	56.7 (18.1-94.4)	57.4 (18.8-91.0)	55.9(18.1-94.4)	***<0.001*** ^*^
**Sex (N, %)**				***<0.001*** ^**^
Female	2,398 (77.8)	1,312 (73.9)	1,086 (83.0)	
Male	686 (22.2)	463 (26.1)	223 (17.0)	
**Tumor type (N, %)**				***<0.001*** ^**^
Breast	1,839 (59.6)	906 (51.0)	933 (71.3)	
Colorectal	524 (17.0)	463 (26.1)	61 (4.7)	
Pancreatic	187 (6.1)	135 (7.6)	52 (4.0)	
Nasopharyngeal	143 (4.6)	82 (4.6)	61 (4.7)	
Glioma	131 (4.2)	48 (2.7)	83 (6.3)	
Gastric	102 (3.3)	38 (2.1)	64 (4.9)	
Biliary	81 (2.6)	42 (2.4)	39 (3.0)	
Ovarian	77 (2.5)	61 (3.4)	16 (1.2)	
**Histology (N, %)**				***0.002*** ^**^
Adenocarcinoma	2,812 (91.2)	1,643 (92.6)	1,169 (89.3)	
Other	272 (8.8)	132 (7.4)	140 (10.7)	
**Stage^†^ (N, %)**				***0.020*** ^**^
Non-metastatic	2,430 (81.0)	1,377 (79.6)	1,053 (83.0)	
Metastatic	569 (19.0)	353 (20.4)	216 (17.0)	
**Grade^†^ (N, %)**				***0.007*** ^**^
Grade 1	165 (5.6)	97 (5.7)	68 (5.4)	
Grade 2	1,300 (43.9)	770 (45.2)	530 (42.2)	
Grade 3	1,299 (43.9)	745 (43.8)	554 (44.1)	
Grade 4	195 (6.6)	90 (5.3)	105 (8.4)	

^†^Data not available for all subjects. Missing values: Age = 14, Grade = 125, Stage = 85.

p-values: ^*^=Wilcoxon rank-sum test, ^**^=Pearson's chi-square test.

Abbreviations: N: number.

### Tumor molecular profiling

**Figure 1:**
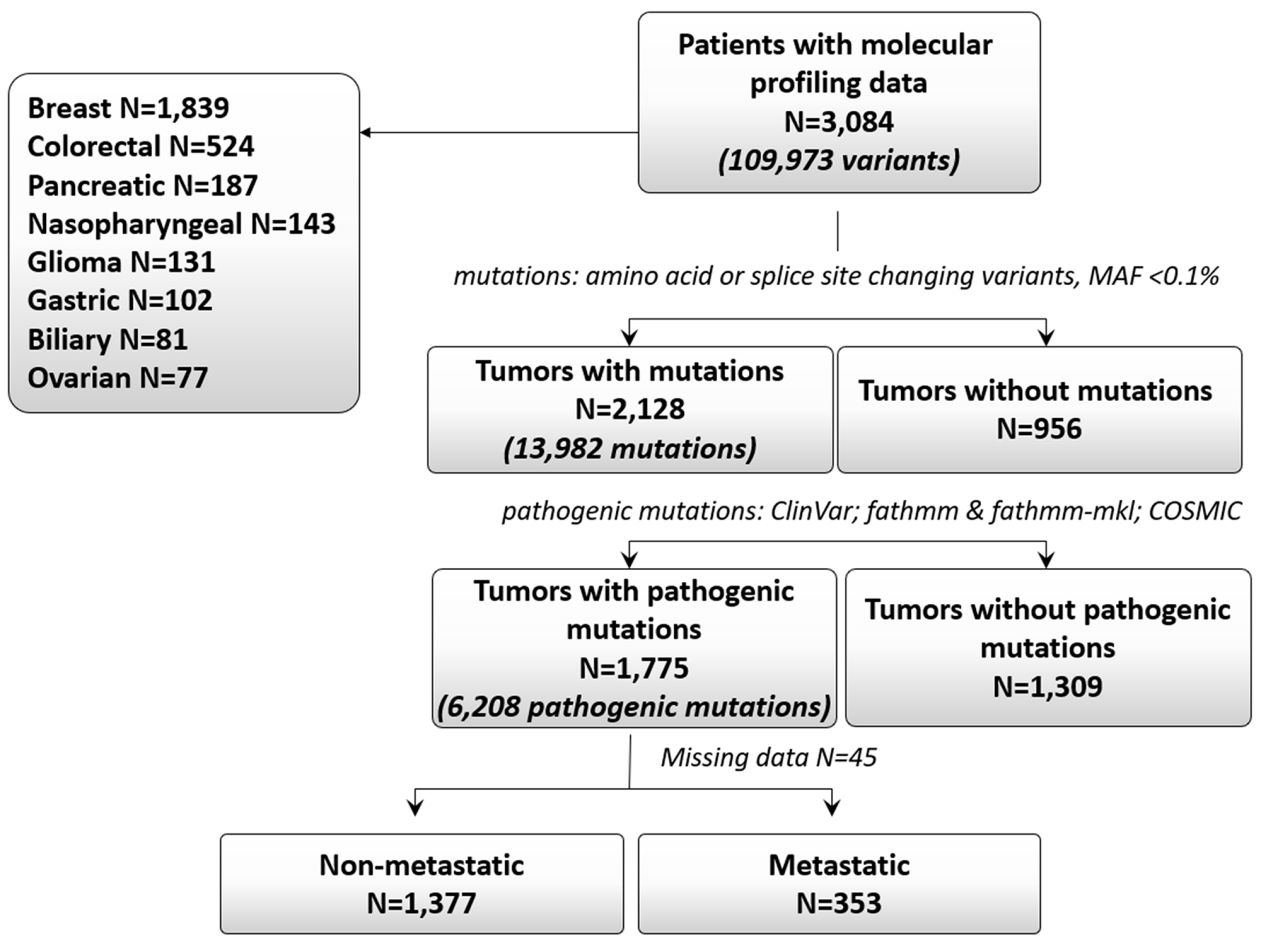
Consort diagram.

**Figure 2:**
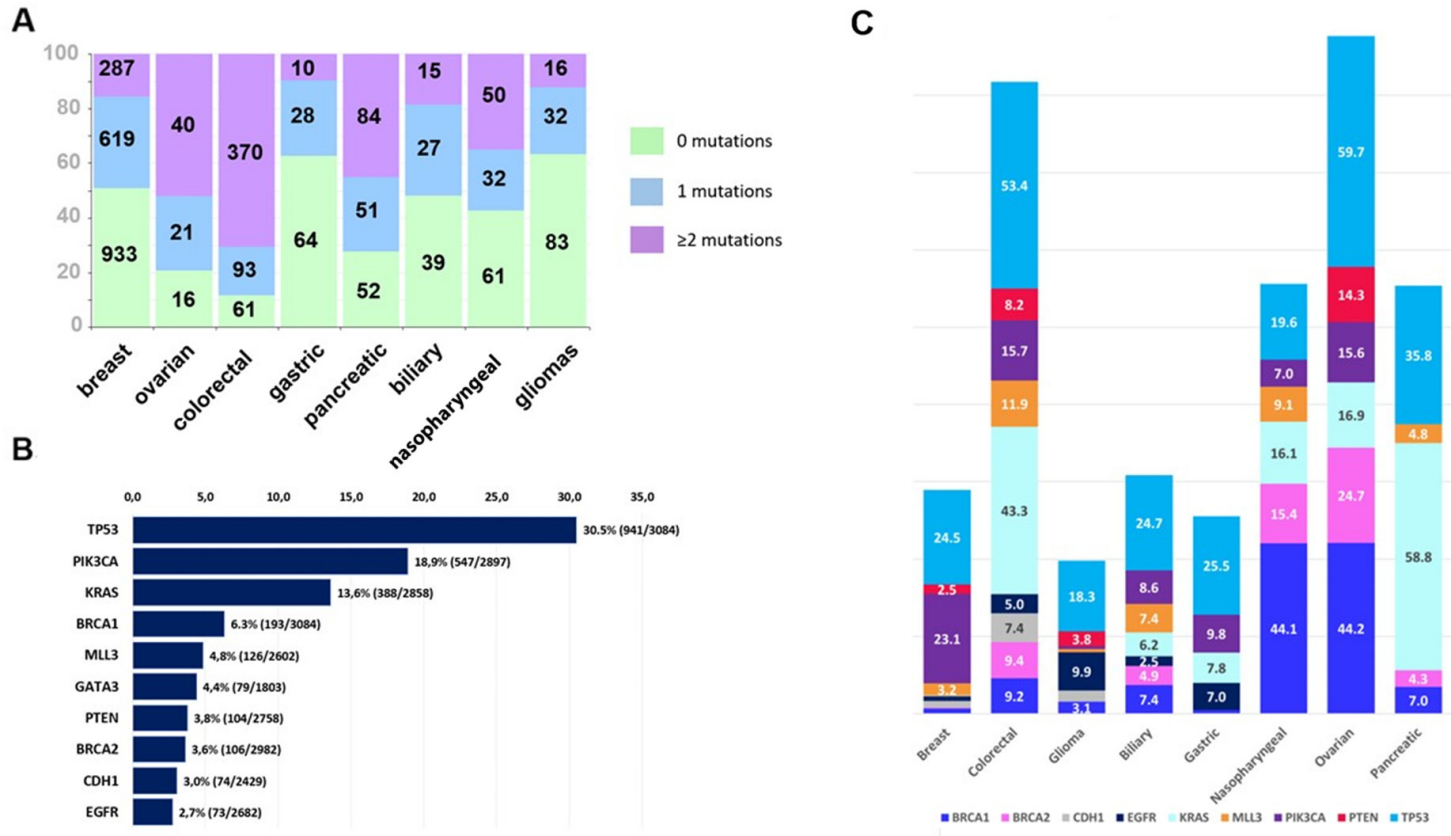
**(A)** Proportions of tumors with 0 (green color), 1 (blue) and ≥2 (purple) pathogenic mutations per tumor type (Kruskal-Wallis p<0.001). Numbers on the bars correspond to the number of tumors. **(B)** Molecular alterations noted in <2.5% of patients tested. Bars indicate proportions of patients whose tumors had a pathogenic mutation (number of tumors with pathogenic mutation/number of tumors tested). Most commonly mutated were *TP53, PIK3CA* and *KRAS* genes. **(C)** Mutation frequencies per tumor type. Bars indicate the proportion of mutated tumors of tumors tested for commonly mutated genes in each of the eight tumor types of the study. Genes included in the graph were tested in ≥75% of patients.

Of the 3,084 patients, 2,128 (69%) patients had tumor mutations. The total number of identified mutations in those 2,128 patients was 13,982. Overall 1,775 (58% of 3,084) patients had pathogenic mutations, including 872 patients who had ≥2 mutations (Table 1, Figure 1). A total of 6,208 pathogenic mutations were identified in those 1,775 patients and were distributed as follows: single nucleotide variants, 88% (n=5,461); stopgains, 10.5% (n=649); frameshifts, 0.9% (n=53); and splice sites, 0.7% (n=45). The proportion of pathogenic mutations by tumor type is shown in Figure 2A. These mutations were most common in the *TP53*, *PIK3CA*, *KRAS*, *BRCA1*, and *MLL3* genes (Figure 2B). The distribution of pathogenic mutations in commonly mutated genes per tumor type is shown in Figure 2C.

### Clinical actionability

Twenty-one genes carried 1687 potentially actionable pathogenic variants. These “actionable genes”, the regions affected within, and the number of affected patients are listed in Supplementary Table 1. Overall, 905 of 3084 patients (29.3%) carried pathogenic tumor variants in these 21 actionable genes. Among these patients, 717 (79.2%) had tumor mutations in 1, and the rest in ≥2 actionable genes. Among the 1687 potentially actionable pathogenic variants, 685 were identified in 13 highly actionable genes and were distributed in the tumors of 403 patients (13.1% of all patients). All highly actionable genes are associated with United States Food and Drug Administration (FDA)-approved therapies (Supplementary Table 1).

### Overall survival

**Figure 3:**
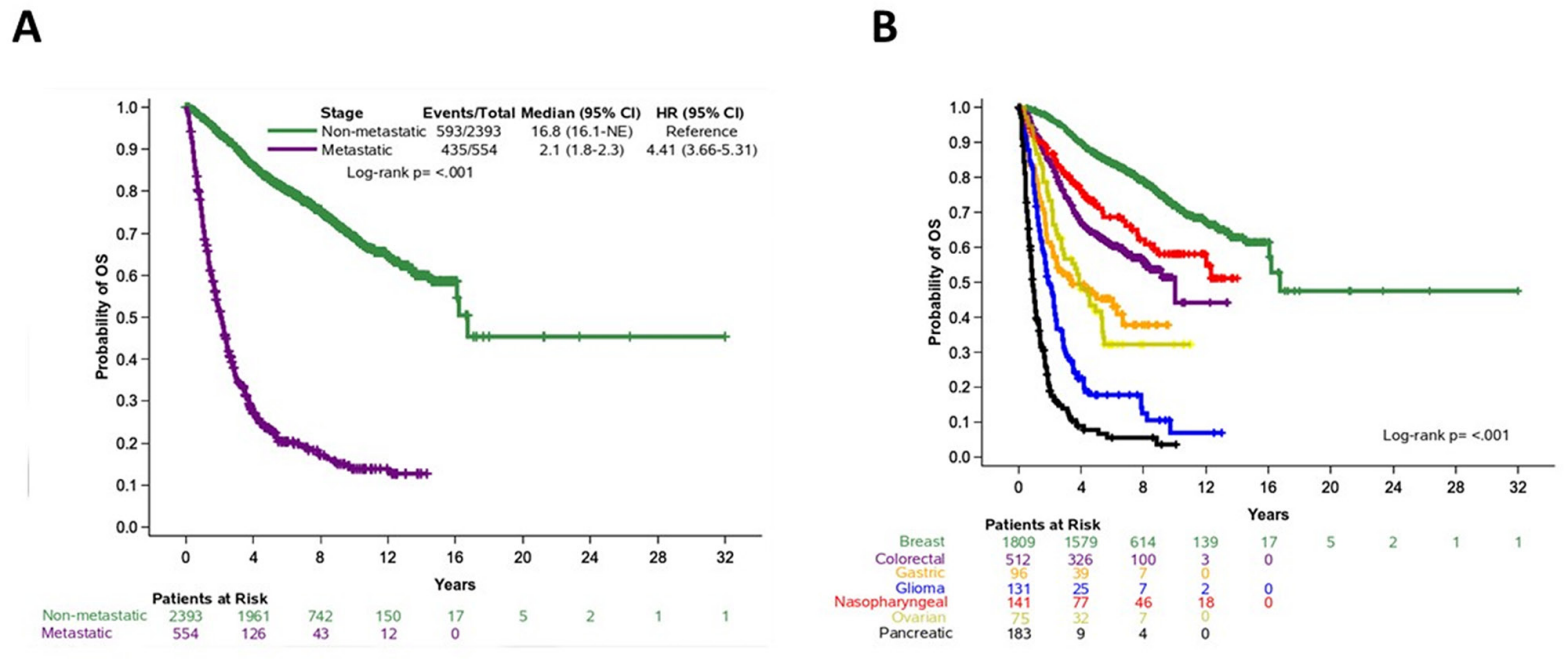
Overall survival in patients based on **(A)** disease stage (non-metastatic and metastatic cancer) and **(B)** tumor type.

The median follow-up of alive patients was 7.52 years (95% confidence interval (CI), 7.39-7.61). Of 2,947 patients with available follow-up data, 1,028 (35%) had died. Of note, follow-up information was not available for patients with biliary tumors. The median OS duration was 16.1 years (95% CI, 12.73-non-reached) for all patients; 16.8 years (95% CI, 16.1-non-reached) for patients with non-metastatic disease; and 2.1 years (95% CI, 1.8-2.3) for patients with metastatic disease (Figure 3A). OS by tumor type is shown in Figure 3B. We performed Cox univariate regression analyses for OS, stratified by tumor type (n=2,947), using the following clinicopathological parameters: age, sex, stage, grade, and histological type. In this analysis, older age, metastatic disease, grade 3-4 disease, and histology other than adenocarcinoma were associated with shorter OS (Supplementary Table 2). Sex was not associated with OS (Wald’s p=0.17).

### Non-metastatic tumors

**Figure 4:**
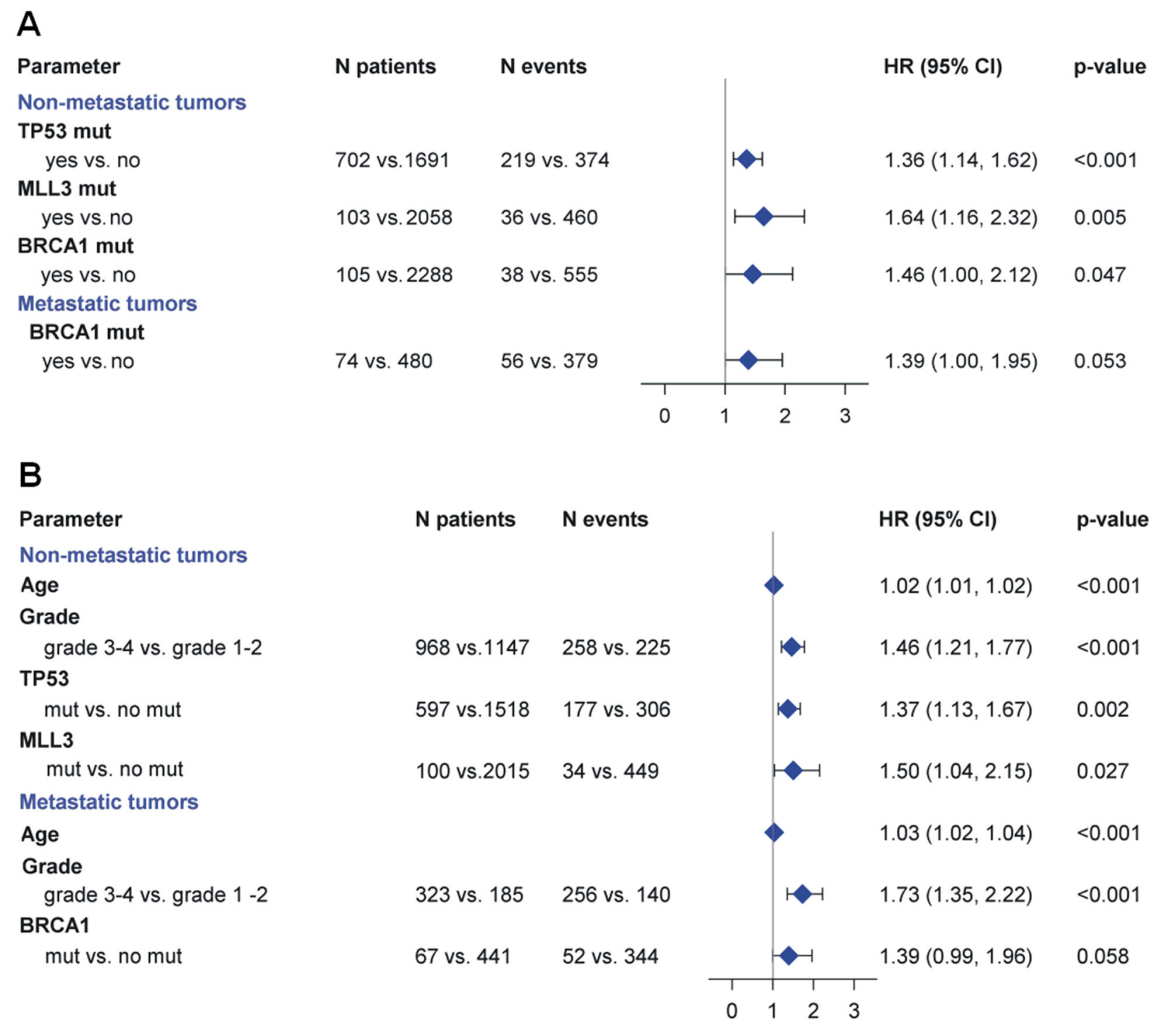
**(A)** Prognostic significance of molecular alterations in patients with non-metastatic and metastatic cancer. In patients with non-metastatic cancer mutations in *TP53*, *MLL3* and *BRCA1* were associated with shorter OS. In patients with metastatic cancer, *BRCA1* mutations were associated with a trend towards shorter OS. **(B)** Multivariate analysis results are depicted in the forest plot. In patients with non-metastatic disease, independent prognostic factors predicting shorter OS were the presence of pathogenic mutations in *TP53*, pathogenic mutations in *MLL3*, increasing age and increasing grade. In the metastatic setting, independent factors associated with shorter OS were increasing age and grade 3-4, while *BRCA1* pathogenic mutations were associated with a trend towards longer OS, after adjustment for age and grade.

Next, we performed univariate analyses after stratification by tumor type in patients with non-metastatic disease. Increasing age, higher grade (grade 3-4) disease, and histology other than adenocarcinoma were associated with shorter OS (Table 2). Subsequently, we assessed the impact of mutations on OS in patients with non-metastatic tumors. OS was shorter in patients with pathogenic *TP53* (Hazard Ratio (HR)=1.36; 95% CI, 1.14-1.62, Wald’s p<0.001), *MLL3* (HR=1.64; 95% CI, 1.16-2.32, p=0.005), and *BRCA1* (HR=1.46; 95% CI, 1.00-2.12, p=0.047) mutations compared to non-mutated genes (Figure 4A).

**Table 2:** Stratified Cox univariate regression with respect to OS in patients with non-metastatic and metastatic tumors

Parameter	N events/Total	HR (95% CI)	p-value
**Non-metastatic tumors**			
**Age^†^**		1.01 (1.01-1.02)	***<0.001***
**Sex**			
Female	438/2001	Reference	--
Male	155/392	1.25 (0.95-1.66)	0.11
**Grade**			
Grade 1-2	258/1231	Reference	--
Grade 3-4	314/1106	1.53 (1.28-1.82)	***<0.001***
**Histology**			
Adenocarcinoma	562/2285	Reference	--
Other	31/108	5.93 (1.18-29.98)	***0.031***
***TP53*** ** mut**			
No	374/1691	Reference	--
Yes	219/702	1.36 (1.14-1.62)	***<0.001***
***MLL3*** ** mut**			
No	460/2058	Reference	--
Yes	36/103	1.64 (1.16-2.32)	***0.005***
***BRCA1*** ** mut**			
No	555/2288	Reference	--
Yes	38/105	1.46 (1.00-2.12)	***0.047***
**Metastatic tumors**			
**Age^†^**		1.03 (1.02-1.03)	***<0.001***
**Sex**			
Female	233/313	Reference	--
Male	202/241	1.15 (0.93-1.42)	0.20
**Grade**			
Grade 1-2	140/185	Reference	--
Grade 3-4	256/323	1.71 (1.35-2.18)	***<0.001***
**Histology**			
Adenocarcinoma	308/392	Reference	
Other	127/162	16.09 (0.97-26.61)	0.053
***BRCA1*** ** mut**			
No	379/480	Reference	--
Yes	56/74	1.39 (1.00-1.95)	0.053

Abbreviations: CI: confidence interval, HR: hazard ratio, mut: mutation, N: number.

^†^ continuous variable.

In multivariate analyses, independent prognostic factors predicting shorter OS were the presence of pathogenic mutations in *TP53* (HR=1.37, 95% CI, 1.13-1.67, p=0.002); pathogenic mutations in *MLL3* (HR=1.50, 95% CI, 1.04-2.15, p=0.027); increasing age (HR=1.02, 95% CI, 1.01-1.02, p<0.001); and increasing grade (HR=1.46, 95% CI, 1.21-1.77, p<0.001). *BRCA1* did not retain its unfavorable prognostic significance for OS (p=0.50) (Figure 4B).

### Metastatic tumors

In univariate analyses, after stratification by tumor type, in patients with metastatic tumors, older age and higher grade were associated with shorter OS, while histology other than adenocarcinoma was associated with a trend towards shorter OS (Table 2). The presence of pathogenic mutations in *BRCA1* (compared to non-mutated *BRCA1*) was also associated with a trend towards shorter OS (Figure 4A). The remaining factors (sex and *TP53*, *MLL3*, and other gene mutations) were not significant.

In multivariate analysis, independent factors associated with shorter OS were increasing age (HR=1.03, 95% CI, 1.02-1.04, p<0.001) and grade 3-4 (HR=1.73, 95% CI, 1.35-2.22, p<0.001). *BRCA1* pathogenic mutations were associated with a trend towards longer OS, after adjustment for age and grade (HR=1.39, 95% CI, 0.90-1.96, p=0.058) (Figure 4B); other factors were not significant.

## DISCUSSION

This is the first comprehensive analysis of NGS data using the database and tumor registry of the HeCOG to assess the association between pathogenic mutations and long-term OS in patients with various tumor types. Previously generated NGS data were evaluated with clinicopathologic and survival data to enable integrative bioinformatic analysis and provide independent prog-nostic biomarkers for OS in the eight tumor types of interest.

Overall, 58% of our patients had pathogenic mutations, most commonly in the *TP53*, *PIK3CA*, *KRAS*, *BRCA1*, and *MLL3* genes. In patients with non-metastatic tumors, pathogenic mutations in *TP53* and *MLL3* were independently associated with shorter OS, along with increasing age and grade. In patients with metastatic tumors, independent factors associated with shorter OS were increasing age and grade. *BRCA1* pathogenic mutations were independently associated with a trend towards shorter OS.

The tumor suppressor gene *TP53* encodes a transcription factor that is activated in response to cellular stress [17, 18]. *TP53* is the most frequently mutated gene in human cancer, and most *TP53* mutations are missense substitutions [19]. Several studies have shown that *TP53* mutations are independent markers of poor prognosis in breast and several other cancers [18, 20–22]. *MLL3*, another tumor suppressor gene, is also mutated in various tumors including glioblastoma, melanoma, and pancreatic and breast cancer. *MLL3* is often deleted in patients with myeloid leukemia and has reduced expression in many breast tumors. In addition, targeted inactivation of *MLL3* in mice leads to epithelial tumor formation. *MLL3* has been associated with decreased OS in patients with diverse tumor types [23, 24]. In our patient group with metastatic cancer, *TP53* and *MLL3* were not associated with prognosis, possibly because of the increasing complexity of mechanisms of cancer evolution in metastatic compared to non-metastatic disease.

Integrative analysis of diverse tumor types (pan-cancer analysis) has been used by several investigators to explore genomic and trascriptomic similarities shared by subgroups of patients across tumor types [5, 6, 25–27]. In one study, the systematic transcriptomic analysis of 6,744 specimens revealed six pan-cancer subnetwork signatures related to cancer cell properties, four of which demonstrated strong prognostic potential [5]. Other investigators performed an integrative analysis of five genome-wide and one proteomic dataset comprising 3,527 specimens from 12 tumor types [6]. They classified tumors into 11 major subtypes on the basis of common molecular alterations, and these classifications reflecting tumor biology were associated with clinical outcomes [6]. Another study reported subgroup-specific clinically relevant gene network characteristics and biological functions based on an integrative pan-cancer genomic analysis of 3,299 samples of 12 tumor types [25]. Finally, analysis of 1,165 exome sequences from 12 tumor types showed that intra-tumor heterogeneity can be used as a universal prognostic biomarker across tumor types [26].

Tumor molecular profiling is increasingly used in the management of patients with cancer. In the randomized SHIVA trial, which assessed molecularly targeted therapy in patients with advanced cancer based on tumor molecular profiling versus conventional therapy, [28] no difference was noted in the primary endpoint of the study (progression-free survival) between the two groups. However, the trial had several limitations [29]. In 2007, the Initiative for Molecular Profiling and Advanced Cancer Therapy (IMPACT) study, a personalized medicine program for patients with advanced cancer, was initiated [30–32]. The study demonstrated that the selection of matched targeted therapy in patients with advanced cancer on the basis of tumor molecular profiling was associated with higher rates of response, progression-free survival, and OS compared to non-matched therapy [32]. Following the example of the IMPACT study, several ongoing clinical trials are evaluating the use of tumor molecular profiling to optimize the selection of targeted therapies across tumor types [33–36].

Tumor NGS has accelerated the development of anticancer therapies by identifying biomarkers predictive of response to targeted treatments. Several studies have assessed the presence of actionable mutations in cancer [37–39]. Although we explored a limited number of genes, we noted a significant proportion of patients (29%) whose tumors harbored at least one pathogenic mutation in a potentially actionable gene. Of these, 45% had pathogenic mutations in highly actionable genes that are associated with FDA-approved therapies. These data may be used to optimize therapy in patients with advanced cancer. However, in patients with metastatic disease, due to tissue availability, NGS was perfomed most commonly in the primary tumor. The presence of actionable molecular alterations in clinical practice should also be assessed in metastatic lesions or through cell-free tumor DNA analysis to account for disease evolution and tumor heterogeneity.

This is the first study of tumor molecular profiling using large-scale NGS data performed in Greece. HeCOG initiated tumor molecular testing in 2013 as a private initiative, without any support from governmental agencies or international networks and consortia. We leveraged the large patient cohort size to conduct a pan-cancer analysis of our data and identify prognostic molecular biomarkers across tumor types. This analysis was empowered by the availability of long follow-up data. This study underlines the importance of the collaboration between several institutions, which led to the collection of thousands of tumor blocks accompanied by detailed clinical data and patient outcomes. This dataset may serve as a valuable resource for the scientific community.

Our study had certain limitations. First, its retrospective nature. Second, tumor molecular profiling was performed with tumor-specific gene panels. Therefore, most of the genes were not assessed in all tumor samples. Additionally, the number of samples differed significantly among tumor types, with breast and colorectal cancers being the most prominent tumor types. Finally, tumors had been selected on the basis of tissue availability, researcher preference, and funding opportunities, possibly introducing selection bias into the analysis.

In conclusion, analysis of molecular data across tumor types can reveal prognostic biomarkers. Taking into consideration the various tumor types included in the analysis, the limited genes analyzed and the heterogeneity of metastatic disease, our data demonstrated that molecular alterations can be used as prognostic biomarkers regardless of the tissue or organ of origin and can improve patient management. Understanding of mechanisms of tumorigenesis and improved therapeutic approaches will lead to improved clinical outcomes.

## MATERIALS AND METHODS

### Patient characteristics

Patients diagnosed with cancer of any tumor type were referred for treatment to academic institutions and private oncology clinics affiliated with HeCOG. All patients received standard-of-care anticancer therapy, and selected patients participated in observational and investigational studies. The patients’ clinical demographic, histopathological, treatment, and outcome data were retrieved from the HeCOG clinical database. The database was established in 1997 and is updated monthly. All patients had provided informed consent for the storage and future use of their biologic materials for research purposes. The research protocol was conducted in agreement with the Declaration of Helsinki and was approved by Bioethics Committee of the participating institutions. The tumor repository was initiated in 1997 to include primarily patients with breast cancer, and it was expanded in 1999 to include patients with additional tumor types, retrospectively and prospectively (registration dates of the oldest samples were as follows: ovarian cancer, 01/1982; nasopharyngeal cancer, 12/1984; colorectal cancer, 08/1988; gastric cancer, 02/1991; breast cancer, 01/1992; biliary cancer, 01/1996; pancreatic cancer, 02/2001; and glioma, 07/2001).

### Tumor samples

FFPE tumor tissue samples from primary (>95% of the cases) and metastatic lesions had been retrieved from the HeCOG tissue repository. Central tumor histologic review; tissue processing, including tissue microarray (TMA) construction, macrodissection, and DNA extraction; NGS genotyping; and initial bioinformatics analysis were performed in the Laboratory of Molecular Oncology, Hellenic Foundation for Cancer Research, Aristotle University of Thessaloniki. Tumor cell content (TCC) corresponded to the rate of tumor cell nuclei versus all nuclei in the tissue area that was processed for DNA extraction. Tumors were used in the present analysis if TCC was <15%.

### NGS genotyping

Genotyping data from 3,084 tumors were retrieved from 14 NGS datasets obtained from 2013 to 2017 in the context of HeCOG translational projects [7–15]. Eight tumor types were genotyped on the basis of tissue sample availability, research interests of the respective investigators, and funding opportunities: breast, colorectal, pancreatic, nasopharyngeal, glioma, gastric, biliary, and ovarian. Details on the NGS method and data processing are provided in Supplementary Materials. Briefly, FFPE DNA was extracted from whole sections or macrodissected tissue fragments or from TMA core sections, quality assessed, and submitted for semiconductor sequencing with 9 custom Ampliseq panels (Thermo-Fisher Scientific, Paisley, UK) that were designed as tumor-specific (e.g., breast, colon) targeting coding regions in 16-101 genes (Supplementary Table 3). The numbers of tumors tested with each panel are shown in Supplementary Table 4. Sequencing results were aligned with Variant Caller versions from 2013 to 2017, annotated with Ion Reporter versions, respectively, and further extensively filtered for ineligible variants on the basis of standard criteria developed at the Laboratory of Molecular Oncology in collaboration with the Victor Chang Cardiac Research Institute, NSW, Australia. Amino acid or splice site changing variants with minor allele frequency <0.1% based on dbSNP, 5000Exomes, and ExAC (for the [non-finnish] European population) were called mutations. The 3,084 tumors analyzed here were selected because they had informative NGS genotypes (single nucleotide variants: position coverage >100; variant coverage >40; indels: position coverage >200; variant coverage >80). The median mean depth was 1,008 (lower quartile: 408; upper quartile: 2,432; range 102.5 – 93,467) (Supplementary Table 5).

### Additional bioinformatics analysis

We further analyzed the NGS data described above using ANNOVAR at the computational genomics laboratory, Department of Genomic Medicine, The University of Texas MD Anderson Cancer Center [40]. We only examined tumor samples without knowledge of patient germline status for cancer predisposing genes. Thus, our samples included both acquired (“somatic”) and potentially inherited (germline) variants. Evidently, identification of variant origin is not accurate when examining tumors only [41]. Therefore, we considered any pathogenic/deleterious variants defined as cancer promoting/driving mutations (e.g., according to COSMIC), irrespectively of their origin. For variants not registered with COSMIC and ClinVar, we used concordant fathmm and fathmm-mkl scores (both deleterious), where available, although pathogenicity prediction in silico with any of the existing tools is far from accurate [42]. Thus, mutations were classified as pathogenic if they fulfilled at least two of the following criteria: ClinVar (pathogenic/likely pathogenic); fathmm score (deleterious); fathmm-mkl (deleterious); COSMIC (pathogenic) [14]. Variants predicted by one fathmm score only were not classified. Mutation classification was performed at the Laboratory of Molecular Oncology, Hellenic Foundation for Cancer Research, Aristotle University of Thessaloniki.

“Clinical actionability” was defined as previously described [39]. Genes were defined as potentially actionable if there was literature supporting clinical benefit in patients with molecular abnormalities in those genes. If the clinical benefit had been reported in any tumor type, the gene was defined as highly actionable, and if there were implications of clinical benefit with a specific treatment, based on the underlying mechanism, the gene was categorized as one that modifies treatment options. However, drug sensitivity is determined by specific molecular abnormalities in a particular gene, while the applied NGS panels for the samples in this study had not been designed to specifically target actionable genomic alterations. For example, ALK fusions (not detected with the applied panels) predict for responsiveness to ALK-inhibitors but mutations in the kinase domain of the same gene (like the ones identified here) usually predict for resistance to the same drugs. Therefore, the present annotation of clinically actionable genes was based on the previously published list, but also on the preset panel targets and on the type of identified pathogenic mutations within specific domains of the previously described actionable genes. Predictive biomarkers (e.g., all RAS genes, ESR1) were not included in the analysis.

### Statistical analysis

Descriptive statistics was used to analyze patients' characteristics. Categorical data, including frequencies and percentages, were described using contingency tables. Continuously scaled measures were summarized by median and range. The association between categorical variables was examined using Pearson’s Chi-square, while comparisons between categorical and continuous variables were examined with the non-parametric Wilcoxon rank-sum test. All tests were two-sided at an alpha 5% level of significance. OS was defined as the time from diagnosis until death from any cause or last follow-up. Survival distributions were estimated using the Kaplan-Meier method; the 2-sided log-rank test stratified by tumor type was used to compare survival between groups.

The proportional hazards assumption was tested for all parameters using time-dependent covariates. Univariate and multivariate Cox proportional hazard regression models were applied to analyze the association of clinicopathological parameters and gene mutational status with death rates. We assessed the prognostic significance of genes assessed in more than 75% of the patients. Analysis was performed separately in patients with non-metastatic and metastatic disease. All models were stratified by tumor type, taking into account the differences between the different types of tumors and the violation of the proportional hazards assumption for this variable. Interaction models, including the product of the stratifying variable with the gene of interest, were examined to evaluate the scenario of obtaining different coefficients for each tumor type. Since no significant interactions between the gene of interest and the stratifying variable were detected using the Wald’s test at the 5% level of significance, we considered the gene effect to not vary across tumor types. To further evaluate the no-interaction assumption, we performed a likelihood ratio test comparing the full (interaction) and the reduced (no-interaction) model. The null hypothesis was that the no-interaction assumption is satisfied, and the test statistic was given by the difference between the log-likelihood statistics of the interaction and no-interaction model [43]. The value of the likelihood ratio statistic was not significant for any of the examined models in metastatic and non-metatastatic patients at the 5% level of significance for the corresponding degrees of freedom. Therefore, the null hypothesis could not be rejected indicating that the no-interaction model should be preferred to the full (interaction) models. Thus, the no-interaction stratified model, assuming same coefficients for each stratum, i.e. tumor type, was considered more appropriate and was applied in our analysis.

In multivariate analyses, a backwards selection procedure with a removal criterion of p>0.10 was applied and included the following clinicopathological parameters in the initial step: age at diagnosis, grade (grade 1-2 vs. grade 3-4), and histological type (adenocarcinoma vs. other), as well as the genes that showed (marginal) statistical significance in the univariate analyses.

No adjustment for multiple comparisons was performed. SAS version 9.3 (SAS Institute) was used for data manipulation and statistical analysis.

## 



## Abbreviations

CI: Confidence interval
FDA: Food and Drug Administration
FFPE: Formalin-fixed, paraffinembedded
HR: Hazard Ratio
HeCOG: Hellenic Cooperative Oncology Group
OS: Overall survival
NGS: Next-generation sequencing
TMA: Tissue microarray
TCC: Tumor cell content
