# Supplementary material for: Pathogenic mutations and overall survival in 3,084 patients with cancer: the Hellenic Cooperative Oncology Group Precision Medicine Initiative
Source: Oncotarget. 2020 Jan 7;11(1):1–14. doi: 10.18632/oncotarget.27338 (PMC6967777; doi:10.18632/oncotarget.27338)
Supplement: Supplementary file 1 [file oncotarget-11-1-s001.pdf]

Pathogenic mutations and overall survival in 3,084 patients with cancer: the Hellenic Cooperative Oncology Group Precision Medicine Initiative

SUPPLEMENTARY MATERIALS

Panels

The scope of genotyping the archived formalin-fixed paraffin-embedded (FFPE) tumors available in the repository of biologic material by the Hellenic Cooperative Oncology Group (HeCOG) was primarily the observation of tumor mutational patterns in the Greek population compared to those described by international consortia, given ethnic differences in cancer characteristics that might relate to targeted treatment selection and efficiency. The herein analyzed 3,084 FFPE tumors had been sequenced at the Laboratory of Molecular Oncology (MOL; Hellenic Foundation for Cancer Research / HeCOG / Aristotle University of Thessaloniki, Greece) over a period of 4 years (2013 – 2017) with 9 custom Ampliseq panels (Supplementary Table 1). Target selection for panel design was mostly based on tumor-type-specific mutations as published by TCGA until the end of 2016, although progressively the panels were enriched to target genes related to DNA repair and immune response. The DNA coordinates targeted by 6 of these panels have previously been published or are in press (available online). This information and the work based on sequencing data by these panels are provided below (*: full description of panel coordinates):

IAD39350_30 (TNBC panel) and IAD47063_31 (breast panel) [1–7] *Clin Breast Cancer, in press; Breast Cancer Res Treat, in press.*

IAD47763_31 (old CRC) [8] *

IAD96775_167 (NPC) [9] * [10]

IAD128757_231 (new CRC) [11] *

IAD75668_167 (ovarian) [12] *

The gene target overlap with these panels is shown below:

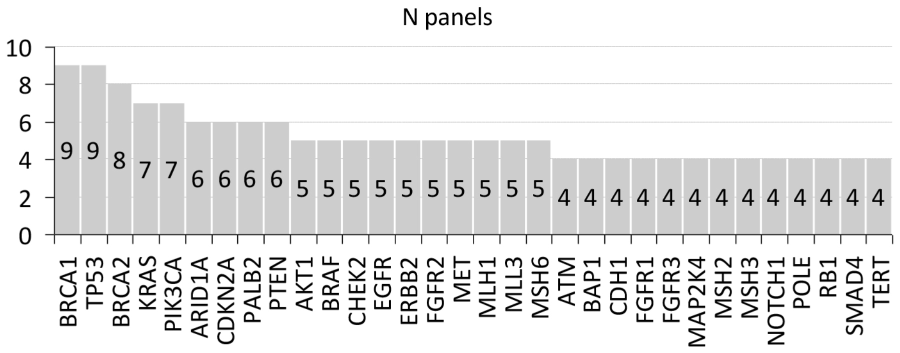

Tissue processing and genotyping

FFPE tissue processing and DNA extraction has been described in the above cited papers. DNA was extracted from whole sections, macrodissected tissue fragments, or tissue microarray cores (TMA) with the QIAamp DNA FFPE Tissue Kit (>90% of the samples) or with the manual version of the VERSANT Tissue Preparation System (Siemens Healthcare Diagnostics. Marburg, Germany) and measured with Qubit (Thermo – Fisher, Waltham, MA). A total of 10 – 15 ng DNA (minimum allowed concentration: 2 ng/ul) was submitted for NGS. All samples were tested with semiconductor sequencing (Ion Proton Sequencer, except for glioma samples that had been run on a Personal Genomic Machine [PGM], by Ion Torrent / Thermo – Fisher) and submitted for alignment to the Ion Torrent server. Variants had been called with the Torrent Suite software v. 3.6.2 – v. 5.6 and annotated with Ion Reporter v. 4 – v. 5.6., initially by using company default plugins. Variants were further stringently filtered out based on the following criteria: p-values (read quality metric) >0.0001 (instead of the preset 0.05 threshold); indels with GC-stretches; min 100 position reads and 40 variant reads for SNVs (min 200 and 80 for indels, respectively); +/- strand read difference >25%; panel-specific mispriming artifacts based on MOL records (e.g., 12:56478880 T>A; 13:32936830 G>A); and variants with allele frequencies (VAFs) <0.12 at positions read <400 times. Overall, samples were classified as failed for mean reading depth <100 and read uniformity <45%, while the minimum number of variants required for sample eligibility was panel specific. Amino acid or splice site changing variants were called mutations for minor allele frequencies <0.1% based on dbSNP, 5000Exomes, and the ExAC project

(http://exac.broadinstitute.org/). This analysis was accomplished for all HeCOG projects at MOL and at the Victor Chang Cardiac Research Institute, Darlinghurst / University of New South Wales, Kensington, NSW, Australia, corresponding to 14 datasets with processed variants (mutations and non-mutations based on the above definition) from multiple matched tissue samples.

Data selection and processing for the present analysis

Mutation data were selected from the above datasets for only one tumor sample per patient, based on best technical characteristics and highest mutation load. The number of tumors tested per panel is shown in Supplementary Table 2 and the technical characteristics of the selected 3,084 tumors are shown in Supplementary Table 3. Genotype data were collated into a new dataset that was submitted for mutation annotation to the computational genomics laboratory, Department of Genomic Medicine, MD Anderson Cancer Center. Out of the returned ANNOVAR parameters, tumor tissue pathogenic mutations were called based on information from COSMIC data and FATHMM scores, as more reliably reflecting the oncogenic potential of tissue variants [13], and ClinVar. Scores pertaining to the evolution of population genetics were avoided.

REFERENCES

1. Kotoula V, Lyberopoulou A, Papadopoulou K, Charalambous E, Alexopoulou Z, Gakou C, Lakis S, Tsolaki E, Lilakos K, Fountzilas G. Evaluation of two highly-multiplexed custom panels for massively parallel semiconductor sequencing on paraffin DNA. PLoS One. 2015; 10:e0128818. https://doi.org/10.1371/journal.pone.0128818. [PubMed]
2. Papaxoinis G, Kotoula V, Alexopoulou Z, Kalogeras KT, Zagouri F, Timotheadou E, Gogas H, Pentheroudakis G, Christodoulou C, Koutras A, Bafaloukos D, Aravantinos G, Papakostas P, et al. Significance of pik3ca mutations in patients with early breast cancer treated with adjuvant chemotherapy: A hellenic cooperative oncology group (hecog) study. PLoS One. 2015; 10:e0140293. https://doi.org/10.1371/journal.pone.0140293. [PubMed]
3. Fountzilas G, Giannoulatou E, Alexopoulou Z, Zagouri F, Timotheadou E, Papadopoulou K, Lakis S, Bobos M, Poulios C, Sotiropoulou M, Lyberopoulou A, Gogas H, Pentheroudakis G, et al. TP53 mutations and protein immunopositivity may predict for poor outcome but also for trastuzumab benefit in patients with early breast cancer treated in the adjuvant setting. Oncotarget. 2016; 7:32731–53. https://doi.org/10.18632/oncotarget.9022. [PubMed]
4. Kotoula V, Lakis S, Vlachos IS, Giannoulatou E, Zagouri F, Alexopoulou Z, Gogas H, Pectasides D, Aravantinos G, Efstratiou I, Pentheroudakis G, Papadopoulou K, Chatzopoulos K, et al. Tumor Infiltrating Lymphocytes Affect the Outcome of Patients with Operable Triple-Negative Breast Cancer in Combination with Mutated Amino Acid Classes. PLoS One. 2016; 11:e0163138. https://doi.org/10.1371/journal.pone.0163138. [PubMed]
5. Fountzilas E, Kotoula V, Zagouri F, Giannoulatou E, Kouvatseas G, Pentheroudakis G, Koletsa T, Bobos M, Papadopoulou K, Samantas E, Demiri E, Miliaras S, Christodoulou C, et al. Disease evolution and heterogeneity in bilateral breast cancer. Am J Cancer Res. 2016; 6:2611–30. [PubMed]
6. Kotoula V, Fostira F, Papadopoulou K, Apostolou P, Tsolaki E, Lazaridis G, Manoussou K, Zagouri F, Pectasides D, Vlachos I, Tikas I, Lakis S, Konstantopoulou I, et al. The fate of BRCA1-related germline mutations in triple-negative breast tumors. Am J Cancer Res. 2017; 7:98–114. [PubMed]
7. Zardavas D, Te Marvelde L, Milne RL, Fumagalli D, Fountzilas G, Kotoula V, Razis E, Papaxoinis G, Joensuu H, Moynahan ME, Hennessy BT, Bieche I, Saal LH, et al. Tumor pik3ca genotype and prognosis in early-stage breast cancer: A pooled analysis of individual patient data. J Clin Oncol. 2018; 36:981–90. https://doi.org/10.1200/JCO.2017.74.8301. [PubMed]
8. Papaxoinis G, Kotoula V, Giannoulatou E, Koliou GA, Karavasilis V, Lakis S, Koureas A, Bobos M, Chalaralambous E, Daskalaki E, Chatzopoulos K, Tsironis G, Pazarli E, et al. Phase II study of panitumumab combined with capecitabine and oxaliplatin as first-line treatment in metastatic colorectal cancer patients: clinical results including extended tumor genotyping. Med Oncol. 2018; 35:101. https://doi.org/10.1007/s12032-018-1160-1. [PubMed]
9. Fountzilas G, Psyrri A, Giannoulatou E, Tikas I, Manousou K, Rontogianni D, Ciuleanu E, Ciuleanu T, Resiga L, Zaramboukas T, Papadopoulou K, Bobos M, Chrisafi S, et al. Prevalent somatic BRCA1 mutations shape clinically relevant genomic patterns of nasopharyngeal carcinoma in Southeast Europe. Int J Cancer. 2018; 142:66–80. https://doi.org/10.1002/ijc.31023. [PubMed]
10. Papadopoulou K, Murray S, Manousou K, Tikas I, Dervenis C, Sgouros J, Rontogianni D, Lakis S, Bobos M, Poulios C, Pervana S, Lazaridis G, Fountzilas G, Kotoula V. Genotyping and mRNA profiling reveal actionable molecular targets in biliary tract cancers. Am J Cancer Res. 2018; 8:2–15. [PubMed]
11. Fountzilas E, Kotoula V, Tikas I, Manousou K, Papadopoulou K, Poulios C, Karavasilis V, Efstratiou I, Pectasides D, Papaparaskeva K, Varthalitis I, Christodoulou C, Papatsibas G, et al. Prognostic significance of tumor genotypes and CD8+ infiltrates in stage I-III colorectal cancer. Oncotarget. 2018; 9:35623–38. https://doi.org/10.18632/oncotarget.26256. [PubMed]
12. Kotoula V, Lakis S, Tikas I, Giannoulatou E, Lazaridis G, Papadopoulou K, Manoussou K, Efstratiou I, Papanikolaou A, Fostira F, Vlachos I, Tarlatzis B, Fountzilas G. Pathogenic BRCA1 mutations may be necessary but not sufficient for tissue genomic heterogeneity: deep sequencing data from ovarian cancer patients. Gynecol Oncol. 2019; 152:375–86. https://doi.org/10.1016/j.ygyno.2018.11.016. [PubMed]
13. Shihab HA, Rogers MF, Gough J, Mort M, Cooper DN, Day IN, Gaunt TR, Campbell C. An integrative approach to predicting the functional effects of non-coding and coding sequence variation. Bioinformatics. 2015; 31:1536–43. https://doi.org/10.1093/bioinformatics/btv009. [PubMed]

Supplementary Table 1: Genes with potentially actionable alterations in the present series

GENE	IMPLICATION (literature)	ACTIONABILITY	potentially actionable mut identified	N potentially actionable mut per gene	N patients with potentially actionable mut	transcript ID (Genebank)	exonic areas with pathogenic mutations
AKT1	Off label or clinical trial of an mTOR/AKT inhibitor	Highly Actionable	YES	51	50	NM_005163.2, NM_001014431.1	ex 2-3
ARID1A	Off label or clinical trial of an mTOR/AKT inhibitor	Modifies Options	YES	24	24	NM_006015.4	ex 2, ex 9-10, ex 15-16, ex 18, ex 20
ATM	Off label or clinical trial of a PARP inhibitor	Highly Actionable	YES	76	48	NM_000051.3	ex 3, 6-7, 9-15, 17, 19-20, 22-25, 27-29, 31, 33-34, 37-43, 45-47, 49-52, 55-56, 58, 60, 62
BAP1	Off label or clinical trial of a PARP inhibitor	Modifies Options	YES	6	6	NM_004656.3	ex 4, 7, 9
BRAF	Off label or clinical trial of a MEK inhibitor	Highly Actionable	YES	78	66	NM_004333.4	ex 11, 15
BRCA1	Off label or clinical trial of a PARP inhibitor	Highly Actionable	YES	363	193	NM_007300.3	ex 2-6, 10-12, 14-24
BRCA2	Off label or clinical trial of a PARP inhibitor	Highly Actionable	YES	152	106	NM_000059.3	ex 2-4, 6-9, 10-11, 13-15, 17-25
CHEK2	Off label or clinical trial of a PARP inhibitor	Highly Actionable	YES	66	49	NM_007194.3, NM_001005735.1	ex 2-6, 9-14
EGFR	Off label or clinical trial of an EGFR inhibitor	Highly Actionable	YES	1	1	NM_005228.3	ex 18
ERBB2	Off label or clinical trial of a HER2-targeting agent	Highly Actionable	YES	36	33	NM_004448.3	ex 5, 7-8, 17, 19-21, 24
FBXW7	Off label or clinical trial of an mTOR/AKT inhibitor	Modifies Options	YES	42	36	NM_033632.3	ex 9-11

(Continued)

GENE	IMPLICATION (literature)	ACTIONABILITY	potentially actionable mut identified	N potentially actionable mut per gene	N patients with potentially actionable mut	transcript ID (Genebank)	exonic areas with pathogenic mutations
FGFR1	Off label or clinical trial of an FGFR inhibitor	Highly Actionable	YES	82	56	NM_001174067.1	ex 5, 10-11, 17-19
FGFR2	Off label or clinical trial of an FGFR inhibitor	Highly Actionable	YES	60	49	NM_000141.4	ex 3, 5, 7, 11, 17-18
FGFR4	Off label or clinical trial of an FGFR inhibitor	Highly Actionable	YES	2	2	NM_002011.3	ex 9
KIT	Off label treatment with a cKIT/PDGFR-targeting agent	Modifies Options	YES	12	11	NM_000222.2	ex 2, 5, 11, 18
PALB2	Off label or clinical trial of a PARP inhibitor	Highly Actionable	YES	22	20	NM_024675.3	ex 3-7
PIK3CA	Off label or clinical trial of an mTOR/AKT inhibitor	Modifies Options	YES	586	547	NM_006218.3	ex 5, 10, 21
POLE	Clinical trial of an immunotherapy or vaccine	Modifies Options	YES	9	9	NM_006231.3	ex 9, 14
RAD50	Off label or clinical trial of a PARP inhibitor	Modifies Options	YES	2	2	NM_005732.3	ex 24
RNF43	Clinical trial of a WNT inhibitor	Modifies Options	YES	2	2	NM_017763.5	ex 9
STK11	Off label or clinical trial of an mTOR/AKT inhibitor	Highly Actionable	YES	15	12	NM_000455.4	ex 8

Supplementary Table 2: Stratified Cox univariate regression with respect to OS for clinicopathological parameters in the entire cohort

Parameter	N events/ Total	HR (95% CI)	p-value
**Age^**		1.02 (1.01-1.02)	***<0.001***
**Sex**			
Female	671/2314	Reference	
Male	357/633	1.13 (0.95-1.33)	0.17
**Stage**			
Non-metastatic	593/2393	Reference	
Metastatic	435/554	4.41 (3.66-5.31)	***<0.001***
**Grade**			
Grade 1-2	398/1416	Reference	
Grade 3-4	570/1429	1.53 (1.53-1.74)	***<0.001***
**Histology**			
Adenocarcinoma	870/2677	Reference	
Other	158/270	7.59 (2.45-23.49)	***<0.001***

Abbreviations: CI: confidence interval, HR: hazard ratio, N: number
^continuous variable.

Supplementary Table 3: Individual tumor series analyzed in the present study; panel characteristics and FFPE samples

HeCOG project	DNA source	Ampliseq Panel ID	Panel description	N amplicons	DNA area targeted	N genes (coding regions)	Year of NGS runs	N analyzed FFPE tissues	N informative FFPE tissues	NGS efficiency (%)	N informative tumors in the present study
BIL	whole sections	IAD47063_31	breast	373	41477	59	2015	162	155	95.7	74
cholangio	whole sections	IAD96775_167	NPC	389	42766	101	2017	84	84	100.0	81
CRC_6C08	TMA cores	IAD128757_231	new CRC	1271	110736	55	2017	464	382	82.3	352
CRC_PM	whole sections	IAD47763_31	old CRC	444	47943	50	2015	177	164	92.7	84
CRC_ vectibix	whole sections	IAD47763_31	old CRC	444	47943	50	2014	119	109	91.6	88
gastric	TMA cores	IAD54638_112	gastric	59	15644	16	2014	182	178	97.8	102
glioma	TMA cores	IAD68363_167	glioma	311	33767	55	2013	156	136	87.2	131
HE1000	whole sections	IAD47063_31	breast	373	41477	59	2014	583	459	78.7	346
HE1005	TMA cores	IAD47063_31	breast	373	41477	59	2014	1016	569	56.0	473
HE1008	TMA cores	IAD47063_31	breast	373	41477	59	2014	763	577	75.6	528
HE1097	whole sections	IAD47063_31	breast	373	41477	59	2014	188	161	85.6	150
NPC	whole sections	IAD96775_167	NPC	389	42766	101	2016	194	145	74.7	143
OV HET	TMA cores	IAD75668_167	ovarian*	330	36820	40	2016	322	297	92.2	74
PAC	TMA cores	WG_ IAD115027.20170228	PAC	339	39894	59	2017	198	188	94.9	187
prophylactic	whole sections	IAD75668_167	ovarian	330	36820	40	2016	210	192	91.4	39
TNBC	TMA cores	IAD39350_30	TNBC	286	29476	41	2013	455	393	86.4	95
trastuzumab	TMA cores	IAD47063_31	breast	373	41477	59	2015	179	176	98.3	137
total samples								***5452***	***4365***		***3084***

* half of this panel targeted common heterozygous SNPs outside gene regions.

Supplementary Table 4: Number of tumors per panel

panel ID	panel description	N tumors	(%)
IAD47063_31	breast	1709	55.4
IAD54638_112	gastric	102	3.3
IAD68363_167	glioma	131	4.2
IAD128757_231	new CRC	352	11.4
IAD96775_167	NPC	224	7.3
IAD47763_31	old CRC	172	5.6
IAD75668_167	ovarian	113	3.7
WG_IAD115027.20170228	PAC	187	6.1
IAD39350_30	TNBC	94	3.0
Total		3084	

Supplementary Table 5: Technical NGS characteristics of tumor DNA samples included in the present analysis

		Mapped Reads	On Target (%)	Mean Depth	Uniformity (%)	Variants
Quantiles						
100.0%	maximum	34100000	98.6	93467.0	98.3	1008
99.5%		8898401	97.9	18204.0	96.6	501
97.5%		4654422	96.9	8889.6	93.8	246
90.0%		2354992	95.4	4679.0	90.9	110
75.0%	quartile	1310190	93.0	2432.0	85.8	42
50.0%	median	511937	87.0	1008.0	80.9	25
25.0%	quartile	229687	80.7	408.0	73.7	19
10.0%		154667	73.8	191.4	57.5	14
2.5%		115165	62.5	127.9	51.3	9
0.5%		102980	52.7	106.7	50.3	5
0.0%	minimum	100198	47.7	102.5	50.0	5
Mean		1020578.4	85.5	1984.2	78.1	49.9
Std Dev		1480067.4	9.0	3242.2	11.6	76.8
N samples		3084	3084	3084	3084	3084
